# Trajectory of individual immunity and vaccination required for SARS-CoV-2 community immunity: a conceptual investigation

**DOI:** 10.1098/rsif.2020.0683

**Published:** 2021-02-03

**Authors:** Chadi M. Saad-Roy, Simon A. Levin, C. Jessica E. Metcalf, Bryan T. Grenfell

**Affiliations:** ^1^ Lewis-Sigler Institute for Integrative Genomics, Princeton University, Princeton, NJ, USA; ^2^ Department of Ecology and Evolutionary Biology, Princeton University, Princeton, NJ, USA; ^3^ Princeton School of Public and International Affairs, Princeton University, Princeton, NJ, USA; ^4^ Division of International Epidemiology and Population Studies, Fogarty International Center, National Institutes of Health, Bethesda, MD, USA

**Keywords:** SARS-CoV-2, natural and vaccinal immunity, community immunity

## Abstract

SARS-CoV-2 is an international public health emergency; high transmissibility and morbidity and mortality can result in the virus overwhelming health systems. Combinations of social distancing, and test, trace, and isolate strategies can reduce the number of new infections per infected individual below 1, thus driving declines in case numbers, but may be both challenging and costly. These interventions must also be maintained until development and (now likely) mass deployment of a vaccine (or therapeutics), since otherwise, many susceptible individuals are still at risk of infection. We use a simple analytical model to explore how low levels of infection, combined with vaccination, determine the trajectory to community immunity. Understanding the repercussions of the biological characteristics of the viral life cycle in this scenario is of considerable importance. We provide a simple description of this process by modelling the scenario where the effective reproduction number $R_{\mathrm{eff}}$ is maintained at 1. Since the additional complexity imposed by the strength and duration of transmission-blocking immunity is not yet clear, we use our framework to probe the impact of these uncertainties. Through intuitive analytical relations, we explore how the necessary magnitude of vaccination rates and mitigation efforts depends crucially on the durations of natural and vaccinal immunity. We also show that our framework can encompass seasonality or preexisting immunity due to epidemic dynamics prior to strong mitigation measures. Taken together, our simple conceptual model illustrates the importance of individual and vaccinal immunity for community immunity, and that the quantification of individuals immunized against SARS-CoV-2 is paramount.

## 1. Introduction

The current COVID-19 pandemic, caused by the SARS-CoV-2 virus, has caused substantial morbidity and mortality across the world [1,2]. This pandemic has additionally led to widespread disruption associated with non-pharmaceutical interventions to mitigate disease burden, notably, social distancing. Historically, the effective deployment of such methods has been successful in temporarily decreasing disease transmission. For example, during the 1918 H1N1 influenza pandemic, Hatchett *et al.* [3] showed that disease burden was substantially reduced in certain US cities due to such widespread measures. Indeed, a comparison between Philadelphia and St Louis illustrates that the latter instituted earlier measures and fared substantially better with a very flat epidemic mortality curve [3]. In the current COVID-19 pandemic, a number of countries have had periods of very flat epidemic curves (e.g. South Korea, Luxemburg, Denmark) due to deployment of non-pharmaceutical interventions. However, this strong level of control has been hard to maintain in most contexts [4]. These flat epidemics could partly be due to better testing, which is an important mitigation measure, in conjunction with contact tracing and social distancing measures.

In infectious disease epidemiology, a key quantity is the basic reproduction number $R_{0}$, defined as the number of new infections that a single infectious individual would infect in a fully susceptible population [5]. According to the simplest mass action SIR models, once the fraction of susceptible individuals is less than $1/R_{0}$, i.e. there is a fraction $H=1-1/R_{0}$ of individuals with immunity or already infected, then an infectious individual would transmit to less than one susceptible individual, and so the disease would die out. Thus, a population with this property has reached *herd or community immunity*, but this threshold can be altered due to factors such as age-specific or other host heterogeneities [5–8]. Herd immunity is salient for mitigation measures in the current COVID-19 pandemic [9,10]. For example, Kissler *et al.* [10] illustrate the interplay between strong mitigation measures and herd immunity. The effective reproduction number $R_{\mathrm{eff}}$ is defined as the basic reproduction number times the fraction of individuals that are susceptible [5], and flat epidemic curves as described above are likely to reflect a scenario where $R_{\mathrm{eff}}$ is close to 1.

In order to curb SARS-CoV-2 transmission, countries are already imposing strong mitigation strategies [11], especially since SARS-CoV-2 can be transmitted to some extent while individuals are pre-symptomatic or asymptomatic or while they show very mild symptoms [12,13]. In practice, numerous countries now exhibit $R_{\mathrm{eff}}$ close to one, due to low but sustained transmission. In this setting, the central theme of additional control measures aimed at long-term population protection is individual immunity, due to natural infection or pharmaceutical interventions, leading to herd immunity. Indeed, in order to attain herd immunity, it is key to optimize the route to vaccinal and natural immunity, and to do so while preventing major transmission will assuredly necessitate pharmaceutical interventions such as vaccination [14] or sera from immune individuals [15]. However, there is currently significant biological uncertainty about the length and strength of natural and vaccinal immunity against the SARS-CoV-2 virus after successful recovery from COVID-19 [16]. Kissler *et al.* [16] explored the role of the duration of immunity in long-term epidemic dynamics, but with many complexities, such as seasonality and multi-strain interactions with existing coronaviruses. Weitz *et al.* [17] examined the role of preferential interactions with immune individuals in epidemic dynamics, and illustrated how this could interact with disease mitigation and herd immunity. With their model, Weitz *et al.* [17] numerically simulated the effect of individual immunity on their results, and highlight that preferential interactions with previously infected individuals can have beneficial effects as long as individual immunity lasts long enough. Furthermore, Kissler *et al.* [10] stress that individual immunity could affect outcomes in the context of social distancing to mitigate disease.

Here, we develop a simple analytical model framework to examine the importance of vaccination rates and the duration of immunity for a trajectory towards community immunity driven by vaccination and a slow accumulation of natural immunity. We first formulate a minimal model of an epidemic without vaccination but under strong non-pharmaceutical mitigation strategies. With our model, in order to eventually attain community immunity, we find an intuitive analytical threshold for the required duration of individual immunity. We then generalize our model to consider the deployment of a vaccine, and investigate the effects of the vaccination rate and the duration of vaccinal immunity on reaching herd immunity, and we find a corresponding relation between individual immunity (either vaccinal or natural) and community protection. Overall, our model is aimed at highlighting conceptual relations between these key quantities, in addition to relative durations. We also examine the consequence of seasonal variation in transmission on the attainment of herd immunity. Lastly, we consider cases where individuals may have preexisting immunity due to epidemic dynamics before mitigation measures began. Overall, while we focus on the COVID-19 disease caused by SARS-CoV-2, our framework is conceptual and can apply to any novel pathogen. Since the quantification of individuals with natural immunity can be achieved through serology, our work underlines the importance of developing appropriate serological assays [18].

## 2. General framework

**Figure 1.**
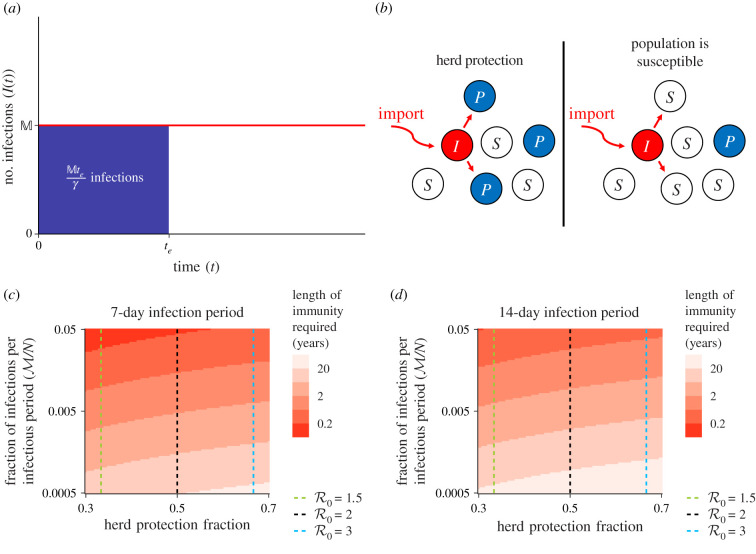
(*a*) Model of *I*(*t*) under disease control so that the epidemic curve is flat. The number of infections that have happened by time *t*_*e*_ is $Mt_{e}/\gamma$, where 1/*γ* is the average infectious period. (*b*) Schematic of a population with herd immunity (left), and susceptible to an outbreak (right) for a basic reproduction number $R_{0}=2$. Here, each circle with *I*, *S* and *P* denotes an individual that is infectious, susceptible or protected (immune). (*c*,*d*) Length of immunity required to reach herd immunity based on the fraction of individuals immune for herd immunity, and the number of infections per infectious period, assuming the infectious period is (*c*) 7 days and (*d*) 14 days. The dashed lines are the homogeneous $1-1/R_{0}$ herd immunity thresholds.

We start with low-level accumulation of natural immunity, and then incorporate vaccination into the framework. To determine the role of individual immunity in population protection, we formulate a minimal model based on a flat epidemic curve. We start by assuming the flattest curve possible, i.e. constant cases through time (equating to $R_{\mathrm{eff}}=1$). Here, we ignore initial transient dynamics, and assume that the system has reached a flat epidemic curve due to control measures such as social distancing, so that the number of infectious individuals at time *t* is I(t)=M (figure 1*a*). Our model only assumes constant incidence as a result of non-pharmaceutical interventions, and makes no assumption on the underlying nature of individual susceptibility and transmission. In particular, superspreading events would not materially affect our calculations given our assumptions.

Omitting births and deaths, if the average duration of infection is 1/*γ*, then it follows that Mt/γ is the total number of infections from 0 to *t*, given that a flat epidemic curve has been reached by time 0. Here, the herd immunity threshold H is reached when individuals in the population are immune or already infected (e.g. figure 1*b* for $R_{0}=2$).

Let *P*(*t*) denote the number of people protected at time *t*. The rate at which individuals become immune is M, measured in individuals per time, so that M=γM. Since the number of infectious individuals is constant over time, M is also the number of infections per time. Further, we assume that the rate of loss of immunity is *δ*. In other words, 1/*δ* is the average time a person is immune to SARS-CoV-2. It follows that

2.1 $$\frac{dP}{dt}=M-\delta P.$$

Integrating this simple equation and assuming that *P*(0) = 0 gives

2.2 $$P(t)=\frac{M}{\delta }(1-e^{-\delta t}).$$

Clearly, as *t* → ∞, P(t)→M/δ  monotonically. Thus, in order to eventually reach herd immunity, it is necessary that the sum of the number of immune individuals and those that are already infected, but not yet immune, is able to attain the number of individuals needed for herd immunity, i.e. it is necessary that (M/δ)+M≥H, which gives

2.3 $$\frac{1}{\delta }\geq \frac{H-M}{M}=\frac{H}{M}-\frac{1}{\gamma }.$$

Thus, interpreting the biological meaning of these parameters, this relation implies that

2.4 $$\mathrm{Length}\;\mathrm{of}\;\mathrm{immunity}\geq \frac{\mathrm{Herd}\;\mathrm{immunity}\;\mathrm{threshold}}{\mathrm{Infections}\;\mathrm{per}\;\mathrm{time}}-\mathrm{Duration}\;\mathrm{of}\;\mathrm{infection}.$$

The relation (2.4) can be understood intuitively. Suppose that natural immunity is lifelong, and that time is measured in durations of infection. *Then, the number of immune individuals would reach the herd immunity threshold when the number of infections per infectious period times the number of infectious periods elapsed is at the herd immunity threshold. Since hosts that are infected but not yet recovered are not susceptible to infection, herd immunity is attained one duration of infection before the number of immune hosts would reach the herd immunity threshold.* If, instead, natural immunity is not lifelong but lasts the length of or longer than this required number of infection periods, then herd immunity is still eventually attainable, but the time until community immunity changes.

Once the epidemic curve is flat, if the length of immunity plus the duration of infection is shorter than the ratio of the number of infections per time to the number of immune individuals needed for herd immunity, then herd immunity through natural infection alone will not stop the epidemic. The basic reproduction number of SARS-CoV-2 is thought to be around 3 (e.g. [19,20]), and so homogeneous models predict that about 67% of the population must be immune or infected to reach herd immunity. The length of natural immunity required for herd immunity under severe control measures is explored in figure 1*c*,*d*.

Another key parameter is the time until the population reaches herd immunity $t_{\;\mathrm{protected}}$. In this simple model, setting P(t)+M=H and solving for *t* gives

2.5 $$t_{\;\mathrm{protected}}=-\frac{1}{\delta }\log\left(1-\delta \left(\frac{H}{M}-\frac{1}{\gamma }\right)\right).$$

**Figure 2.**
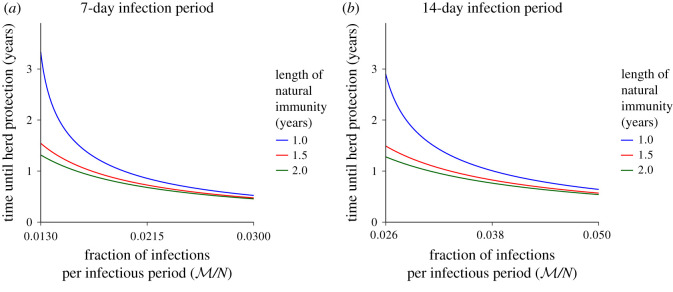
Time until herd immunity is reached as a function of the fraction of infections per infectious period (M/N) and the length of natural immunity (1/*δ*). Here, we assume that the herd immunity threshold is H/N=2/3, corresponding to $R_{0}=3$, and we compute $t_{\;\mathrm{protected}}$. As in figure 1, (*a*) and (*b*) depict a 7- and 14-day infection period, respectively. Note that the scale of the *x*-axis between panels (*a*) and (*b*) changes to allow for proper *y*-axis comparisons.

As an illustration, in figure 2 we compute the time until protection for different average lengths of natural immunity, across a range of M/N, where *N* is the total population size, i.e. M is normalized. Since the average length of conferred immunity after SARS-CoV-2 infection is presently unknown, we schematically present in figure 2 the shape of these curves with natural immunity persisting for on average 1–2 years. Here, we find that the time until herd immunity can be smaller than a year with high enough infection. In other cases, herd immunity can be attained within a few years. Intuitively, for faster immune waning (i.e. larger *δ*), our model predicts that the time until protection is longer.

For certain parameter values in our model, the times to reach herd immunity qualitatively agree with time estimates (less than a year) obtained by Kissler *et al.* [10] for herd immunity under strong and long social distancing; however, these authors did not consider waning immunity in their models, but they mention that waning immunity would affect their results. Indeed, waning immunity increases the time until population protection, especially if waning is rapid, and our model highlights this point.

Reaching the herd immunity threshold is important to allow for the eventual relaxation of strong mitigation strategies such as extreme social distancing. However, as proposed by Weitz *et al.* [17], social distancing could be decreased progressively for individuals that have attained immunity. In this scenario, reaching the vicinity of the original herd immunity threshold could still lead to an ‘effective’ population protection. Thus, if we instead suppose that fH of the population has to be immune or infected, then analogous relations hold. Indeed, relation (2.3) becomes

2.6 $$\frac{1}{\delta }\geq \frac{\;fH}{M}-\frac{1}{\gamma }$$

and the time until ‘effective’ protection is

2.7 $$t_{\;p}^{\left(f\right)}=-\frac{1}{\delta }\log\left(1-\delta \left(\frac{\;f\;H}{M}-\frac{1}{\gamma }\right)\right).$$

The dependence of $t_{\;p}^{\left(f\right)}$ on *f* is

2.8 $$\frac{\partial t_{\;p}^{\left(f\right)}}{\partial f}=\frac{H}{M}\;e^{\delta t_{\;p}^{\left(f\right)}}>0.$$

Thus, intuitively, decreasing *f* decreases the time until protection, and the relative advantage of a decrease in *f* depends upon the other parameter values. As a special case, we present an application of our framework in electronic supplementary material, where the number of infections is limited by the number of ICU beds (see An application with ICU beds, electronic supplementary material, and figure S1).

## 3. Introducing vaccination

Numerous vaccines against SARS-CoV-2 are currently in development. Eventually, a vaccine will hopefully be readily available, and our framework gives insight into the number of individuals successfully immunized by vaccination required to attain herd immunity. The deployment of transmission-reducing vaccination can have two roles in population immunity: (1) it can be the only approach to reach herd immunity, e.g. in a scenario where natural infections are rare, and (2) it can decrease the time until population protection.

We first make the pessimistic assumption that natural immunity is sufficiently transient so that natural immunity alone cannot lead to herd immunity, i.e. 1/δ<H/M−1/γ. Since the number of individuals with natural immunity against the virus approaches $\lim_{t\to \infty }P(t)=M/\delta$, then it follows that the steady state is M/δ=γM/δ. Therefore, in this setting, assuming that vaccinal immunity is effective, the number of individuals that require successful immunization from a vaccine is H−M(1+γ/δ).

**Figure 3.**
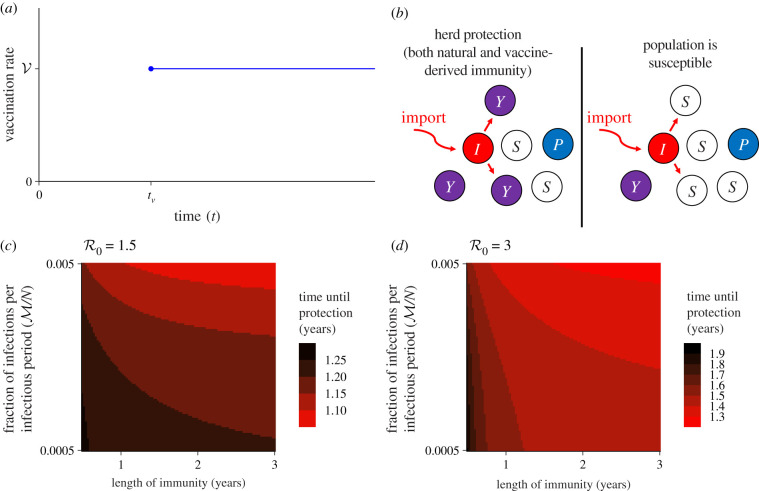
Introduction of vaccination into the simple immunity model. (*a*) Vaccination is assumed to start at time *t*_*v*_ and occur at a constant rate V. (*b*) An illustrative schematic of herd immunity through natural infection and vaccination for $R_{0}=2$, where *S* denotes a susceptible individual, *P* and *Y* denote individuals with natural and vaccine-derived immunity, respectively, and *I* denotes the initial infectious individual (cf. figure 1*a*). Note that on the left, since $R_{0}=2$, more individuals have immunity than required for herd immunity (4/6 versus 3/6, respectively). (*c*,*d*) Illustrative schematics of the interplay between natural immunity and vaccine-derived immunity in order to reach herd immunity. In both panels, it is assumed that vaccination starts at *t*_*v*_ = 1 year, that the infection period is 7 days, that the rate V=0.03 per infection duration, and that *ρ* = *δ*. The number of individuals with immunity required for herd immunity depends upon $R_{0}$: (*c*) $R_{0}=1.5$; (*d*) $R_{0}=3$.

To incorporate more nuance, suppose that immunity from vaccination of individuals without natural immunity occurs at a constant rate V and starts at time *t*_*v*_ (figure 3*a*), so that herd immunity could eventually be reached through both vaccination and natural immunity (figure 3*b*). The assumption of a constant vaccination rate is feasible, because there are bottlenecks in vaccine development and deployment that could reasonably lead to a constant number of individuals getting vaccinated per time. We also assume that vaccine-derived immunity wanes at rate *ρ*, which could be different from the rate of waning for natural immunity *δ*. Then, the number of individuals with vaccine-derived immunity *Y*(*t*) at time *t* follows

3.1 $$\frac{dY}{dt}=V-\rho Y.$$

Since vaccination is started at time *t*_*v*_, it follows by integration that

3.2 $$Y(t)=\left\{ \begin{array}{cc} 0\; & t<t_{v}\\ \frac{V}{\rho }(1-e^{-\rho (t-t_{v})}) & t\geq t_{v}. \end{array}\right.$$

Thus, the total number of individuals with immunity, either from natural infection or from vaccination, is *T*(*t*) = *P*(*t*) + *Y*(*t*), so that

3.3 $$T(t)=\left\{ \begin{array}{cc} \frac{M}{\delta }(1-e^{-\delta t})\; & t<t_{v}\\ \frac{M}{\delta }(1-e^{-\delta t})+\frac{V}{\rho }(1-e^{-\rho (t-t_{v})}) & t\geq t_{v}. \end{array}\right.$$

Again, since the goal is to reach herd immunity, we assume that this cannot be reached before the start of vaccination. We note that *T*(*t*) is monotonically increasing in time, and so herd immunity can be achieved if the number of protected individuals and those previously infected can reach the number of individuals required for herd immunity, and we set $H=(M/\delta )(1-e^{-\delta t})+(V/\rho )(1-e^{-\rho (t-t_{v})})$. Since we assume that herd immunity is not attained at *t*_*v*_, then *t* > *t*_*v*_ and so the right-hand side is monotonically increasing, it follows that herd immunity is eventually reached if and only if H≤M/δ+V/ρ+M. This inequality can be simply rearranged to give

3.4 $$\frac{1}{\delta }+\frac{1}{\gamma }\geq \frac{H}{M}-\frac{1}{\rho }\frac{V}{M}.$$

Thus, the length of natural immunity plus the period of infection has to be equal to or greater than the difference between the corresponding threshold without vaccination and the ratio of the vaccination rate to infections weighted by the length of vaccine-derived immunity.

Another important goal of vaccination is to reach herd immunity more rapidly than from natural infection, thus averting a substantial number of infections. If vaccine-derived and natural immunity last for similar time periods, i.e. *ρ* ≈ *δ*, then, assuming herd immunity is not reached through natural immunity alone by *t*_*v*_, the time until herd immunity is

3.5 $${\begin{array}{r} t \end{array}}_{\;\mathrm{protected}}=-\frac{1}{\delta }\log\left[1-\delta \left(\frac{H}{M}-\frac{1}{\gamma }\right)+\frac{V}{M}\right]+\frac{1}{\delta }\log\left[1+\frac{V}{M}e^{\delta t_{v}}\right].$$

The dependence of the time until protection $t_{\;\mathrm{protected}}$ on the time that vaccination is initiated *t*_*v*_ is $\partial t_{\;\mathrm{protected}}/\partial t_{v}=V/(M\;e^{-\delta t_{v}}+V)$. Further, if M≪V, then $\partial t_{\;\mathrm{protected}}/\partial t_{v}\approx 1$, i.e. an increase in the time of vaccination initialization leads to a corresponding increase in the time until protection. This is intuitive, since the assumption M≪V implies that the number of individuals obtaining immunity through infection is negligible as compared to those undergoing vaccination. Here again, by replacing H by fH, we obtain the time until a fraction *f* of the herd immunity threshold has been reached.

In figure 3*c*,*d*, we schematically illustrate how the length of immunity can impact the time until a population reaches herd immunity for different regimes of constant infection levels. Under equivalent vaccination efforts, if herd immunity requires a larger fraction of immune individuals (i.e. smaller versus bigger $R_{0}$, figure 3*c* versus figure 3*d*), then the dependence of the time until community immunity on the duration of individual immunity is greater. Intuitively, this observation follows since a larger fraction for herd immunity is reached through a greater relative fraction of individuals with natural immunity.

## 4. Incorporating seasonality

Disease transmission can vary throughout time, often on seasonal timescales (due to, for example, climatic drivers and/or population aggregation in schools; e.g. [21–23]). The inclusion of seasonality in our framework could also represent a very simple model of a mitigation strategy that oscillates between two regimes. Intrinsic seasonality could affect the basic reproduction number and the herd immunity threshold, and these effects have been examined through mathematical models with periodicity [24–26]. Here, we assume that the herd immunity threshold H is known.

To model seasonality, we consider two constant values for the number of infectious individuals at any given time (electronic supplementary material, figure S1), so that I(0)=M and

4.1 $$I(t)=\left\{ \begin{array}{cc} M & 2nt_{s}<t\leq (2n+1)t_{s}\;\\ W & (2n+1)t_{s}<t\leq (2n+2)t_{s}, \end{array}\right.$$

where *t*_*s*_ is the time for half a period and *n* is a measure of the number of periods.

The rate of infections per time in the second half of a period is W, so that W=γW. Thus, the number of individuals that have individual immunity follows

4.2 $$\frac{dP}{dt}=\left\{ \begin{array}{cc} M-\delta P & 2nt_{s}<t\leq (2n+1)t_{s}\;\\ W-\delta P & (2n+1)t_{s}<t\leq (2n+2)t_{s}. \end{array}\right.$$

The details of the calculation to obtain *P*(*t*) are omitted here but are included in electronic supplementary material, and these give that

4.3 $$P(t)=\left\{ \begin{array}{rl} & \left(\frac{W-M}{\delta }\right)(1-e^{-\delta t_{s}})\sum_{k=1}^{n}\;e^{-\delta (t-2kt_{s})}\\ & \;\;+\frac{M}{\delta }(1-e^{-\delta t}),2nt_{s}\leq t\leq (2n+1)t_{s}\\ & \left(\frac{W-M}{\delta }\right)(1-e^{-\delta t_{s}})\sum_{k=1}^{n}\;e^{-\delta (t-2kt_{s})}\\ & \;\;+\left(\frac{M-W}{\delta }\right)e^{-\delta (t-(2n+1)t_{s})}\\ & \;\;+\frac{W}{\delta }-\frac{M}{\delta }\;e^{-\delta t},(2n+1)t_{s}\leq t\leq (2n+2)t_{s}, \end{array}\right.$$

where the sum from *k* = 1 to *n* is zero if *n* = 0. After *n* periods, *t* = 2*nt*_*s*_, and *P*_*n*_ = *P*(2*nt*_*s*_). *P*_*n*_ is a monotonically increasing sequence of *n* (see electronic supplementary material for details), and taking the limit of *P*_*n*_ as *n* → ∞ gives

4.4 $$\lim_{n\to \infty }P_{n}=\frac{M}{\delta }-\left(\frac{M-W}{\delta }\right)\frac{e^{t_{s}\delta }}{e^{\delta t_{s}}+1}.$$

Here, *I*(*t*) is discontinuous at 2*nt*_*s*_ and (2*n* + 1)*t*_*s*_, and this discontinuity introduces a difficulty for the computation of *I*(*t*) + *P*(*t*) at the end of a period. Thus, after a certain number of periods, we instead take the sufficient condition that the number of immune individuals can reach the number of individuals needed for herd immunity H. That is, since *P*_*n*_ is monotonically increasing, if $\lim_{n\to \infty }P_{n}\geq H$ then H can be attained. Thus, the duration of immunity 1/*δ* must satisfy

4.5 $$\frac{1}{\delta }\geq \frac{H}{M-(M-W)(e^{\delta t_{s}}/(e^{\delta t_{s}}+1))},$$

since $\lim_{n\to \infty }\sum_{k=1}^{n}\;e^{-2\delta t_{s}(n-k)}=e^{2t_{s}\delta }/(e^{2t_{s}\delta }-1)$ and $e^{2t_{s}\delta }-1=$ $\left(e^{\delta t_{s}}-1)(e^{\delta t_{s}}+1\right)$. Equation (4.5) is a transcendental equation for *δ* due to the presence of $e^{\delta t_{s}}$, and (4.5) also depends on the number of infections that occur during each half period (M and W) and the length of half a period *t*_*s*_.

If M>W (as assumed in electronic supplementary material, figure S1), it is intuitive that the corresponding limit of fraction protected after offset periods should be greater than the value given above. Furthermore, in this case, it is easy to see that P(t)≤M/δ for 2*nt*_*s*_ ≤ *t* ≤ (2*n* + 1)*t*_*s*_, which implies that *P*(*t*) is increasing on this interval. Thus, the maximal fraction of protected individuals on each such interval is at the end of an offset period, i.e. at *Q*_*m*_ = *P*((2*m* + 1)*t*_*s*_). *Q*_*m*_ is a monotonically increasing sequence of *m* by a similar argument as for *P*_*n*_, and

4.6 $$\lim_{m\to \infty }Q_{m}=\frac{M}{\delta }-\left(\frac{M-W}{\delta }\right)\frac{1}{e^{\delta t_{s}}+1}.$$

In this scenario, since again *I*(*t*) is discontinuous at *t* = (2*m* + 1)*t*_*s*_, we focus on the immune individuals. At an offset period, a sufficient condition for herd immunity is if the limit as *m* → ∞ of the number of immune individuals reaches or exceeds the herd immunity threshold. This condition gives the transcendental equation

4.7 $$\frac{1}{\delta }\geq \frac{H}{M-(M-W)(1/(e^{\delta t_{s}}+1))}.$$

Here, considering vaccination at a constant rate V at time *t*_*v*_ > 0 can be incorporated akin to the model without seasonality. As previously, the number of individuals with vaccinal immunity that wanes at rate *ρ* is $Y(t)=(V/\rho )(1-e^{-\rho (t-t_{v})})$ and the total number of individuals with natural or vaccinal immunity is *T*(*t*) = *P*(*t*) + *Y*(*t*) also.

## 5. Preexisting immunity

So far, we have assumed that no individuals have immunity at *t* = 0. However, depending on the timing of mitigation measures, it is possible that there is an initial transient that does not follow the flat curve, which gives rise to individuals that are immune before our model applies. For example, during the current COVID-19 pandemic, South Korea had an initial increase in cases, before a subsequent decrease to a flat incidence curve. Our framework can be easily adapted to incorporate preexisting immunity. Indeed, we can generalize the model to include *P*(0) = *P*_0_ individuals with immunity at *t* = 0 when the flat line begins, where *P*_0_ is not at or beyond herd immunity and is such that $P_{0}<M/\delta$. Thus, the number of protected individuals over time is then $P(t)=(M/\delta )(1-e^{-\delta t})+P_{0}\;e^{-\delta t}$. Since lim _*t*→∞_*P*_0_ e^−*δt*^ = 0, and *P*_0_ is assumed less than M/δ, relation (2.4) on the duration of immunity required to reach herd immunity does not change, but the time until protection is now

5.1 $$t_{\;\mathrm{protected}}=\frac{1}{\delta }\log\left[1-\frac{\delta P_{0}}{M}\right]-\frac{1}{\delta }\log\left[1-\delta \left(\frac{H}{M}-\frac{1}{\gamma }\right)\right]$$

(otherwise, if $P_{0}>M/\delta$, then the maximal value of *P*(*t*) is *P*_0_).

With vaccination, the total number of individuals with natural or vaccinal immunity is

5.2 $$T(t)=\left\{ \begin{array}{cc} \frac{M}{\delta }(1-e^{-\delta t})+P_{0}\;e^{-\delta t}\; & t<t_{v}\\ \frac{M}{\delta }(1-e^{-\delta t})+\frac{V}{\rho }(1-e^{-\rho (t-t_{v})})+P_{0}\;e^{-\delta t} & t\geq t_{v}. \end{array}\right.$$

Here again, provided $P_{0}<M/\delta$ and since lim_*t*→∞_*P*_0_ e^−*δt*^ = 0, relation (3.4) does not change. Under the further assumption that natural and vaccinal immunity wane at approximately the same rate, i.e. *δ* ≈ *ρ*, and that herd immunity is not reached by the time vaccination is introduced, the time to protection becomes

5.3 $$t_{\;\mathrm{protected}}=-\frac{1}{\delta }\log\left[1-\delta \left(\frac{H}{M}-\frac{1}{\gamma }\right)+\frac{V}{M}\right]+\frac{1}{\delta }\log\left[1+\frac{V}{M}\;e^{\delta t_{v}}-\delta \frac{P_{0}}{M}\right].$$

Lastly, with seasonality, a similar extension also follows.

## 6. Discussion and conclusion

We investigated the relationship between vaccination, individual and vaccinal immunity, community immunity, and the time until a population is successfully protected, which are all key variables for successful disease mitigation of the current COVID-19 pandemic. We formulated a simple conceptual model of an epidemic, motivated by very flat epidemic curves that can occur under adequate non-pharmaceutical interventions, such as seen in St Louis during the 1918 H1N1 pandemic [3]. In a nutshell, the pillar of our analyses centres on achieving community immunity against SARS-CoV-2 through individual vaccinal and natural immunity in a setting where transmission is restricted to a low level of infections.

Here, we have shown that the timescale of individual immunity, whether natural or vaccinal, is important to reach community immunity. We first simply portray the important relationship between individual and population immunity by considering a model without vaccination and a constant number of infectious individuals. In this setting, in order for the population to reach the threshold for herd immunity, we analytically derived that the duration of individual immunity plus the infectious period has to be equal to or greater than the ratio of the herd immunity threshold to the number of infections at any given time. In the case that herd immunity is eventually reached, we also analytically calculated the time until the population is protected. We assume that $R_{\mathrm{eff}}=1$ for analytical simplicity, which is conservative and captures the essence of the interactions between individual and herd immunity. More complete control ($R_{\mathrm{eff}}<1$) would decrease accumulation of immunity.

While natural infection leads to individual immunity, pharmaceutical interventions can also protect individuals, such as vaccination through vaccinal immunity. We therefore incorporated the introduction of a vaccine and subsequently vaccination at a fixed rate in our model. Here, we obtained an analogous inequality on the duration of natural immunity for herd immunity as a function of the length of vaccine-derived immunity and vaccination rate. Furthermore, if vaccinal and natural immunity wane at approximately the same rate, we computed the time until a population is protected. We find that the dependence of this duration on the time when a vaccine is introduced is determined by the duration of immunity, the magnitude of the vaccination rate and the time a vaccine is introduced.

We then investigated the effect of ‘seasonality’, either due to intrinsic properties of the disease or the environment, or as the result of an oscillatory control strategy, on community immunity by incorporating simple periodicity. With this extended model, we derived analogous conditions that, in the long run, guarantee eventual herd immunity at the end of a half-period or full period. Lastly, we incorporated individuals with preexisting immunity to model situations with an initial transient period before control measures are enacted and a flat epidemic curve is reached.

To distill the importance of the duration of individual immunity and vaccination on the epidemic trajectory and their relation to community immunity, we have formulated a simplified framework that incorporates these key components but omits other realistic factors. For example, by assuming a constant influx of infections, we have ignored more complex transmission dynamics. Yet, for certain parameter values, our framework captures the approximate time until herd immunity obtained through more realistic simulations with lifelong immunity [10]. Furthermore, we have omitted specific biological details, such as the natural deaths of immune individuals, in our analyses. Mortality of individuals that are immune only affects the effective rate of waning immunity in our framework. Indeed, if mortality is considered, the effective rate of waning immunity is the sum of the actual waning rate and the natural mortality rate. Thus, with natural deaths, the relations we derived would instead be for the death-adjusted length of immunity rather than purely for the average duration that a host is immune. As we have illustrated, our conceptual model can be further refined for specific applications and increased biological realism. Indeed, we also omit many other complexities, e.g. imperfect immunity [27,28], which are important areas for future work. Additionally, while we have incorporated vaccination into our framework, we have assumed that the herd immunity threshold is independent of both the relative level of vaccination to infection incidence and of the vaccination strategy. However, population heterogeneity can affect the infection herd immunity threshold [5–7], and the type of vaccination (random versus directed) in the presence of heterogeneity can alter the vaccination herd immunity threshold [5,8]. Incorporating these specific considerations into our framework of constant incidence would be valuable.

Overall, from a population perspective, our work underlines how the attainment of population protection is interconnected to the rate at which individuals are vaccinated in addition to the duration of individual immunity, whether natural or vaccinal. We also illustrate that our model can encompass other relevant biological factors. The inclusion of these factors gives conceptual characterizations of their impacts on the intuitive relations that tie vaccination, individual immunity and community immunity. Due to the centrality of individual immunity, it is important to quantify individuals immune against SARS-CoV-2, and it is crucial to determine the strength and duration of both vaccinal and natural immunity. Such a characterization could be achieved through serology [18]. While we emphasize the current COVID-19 pandemic, the analytical framework we developed is general and could be used with other pathogens against which a combination of vaccination and strong mitigation measures are applied.

## Data accessibility

This article has no additional data.
